# Bilateral Acute Retinal Necrosis Treated With Antivirals and Corticosteroids

**DOI:** 10.7759/cureus.47427

**Published:** 2023-10-21

**Authors:** Estefania Ramirez Marquez, Alejandra Santiago, Israel Mendez, Jan C Santiago, José J López-Fontanet, Noraliz Garcia, Armando L Oliver

**Affiliations:** 1 Ophthalmology, University of Puerto Rico School of Medicine, Medical Sciences Campus, San Juan, USA

**Keywords:** antivirals, corticosteroids, herpes, retina, uveitis, arn, barn

## Abstract

We report on the case of a Hispanic male with bilateral acute retinal necrosis (BARN), whose disease was managed with intravenous acyclovir, topical prednisolone, topical atropine, intravitreal ganciclovir, oral valacyclovir, and oral prednisone. The oral corticosteroid was added to his treatment regimen eight days after his initial presentation. The 55-year-old patient presented with a two-week history of bilateral blurred vision. His medical history was pertinent for remote varicella and herpes zoster (the latter limited to the forehead). His best-corrected visual acuity (BCVA) was counting fingers in both eyes (OU). His examination was remarkable in OU for grade 3+ cells, pharmacologically dilated pupils, and grade 2+ vitreous cells. The patient's fundus was remarkable in OU for optic-nerve swelling, vitreous condensation, ghost vessels, and retinitis patches. Given the clinical and ancillary testing results, an assessment of BARN was made. The patient received acyclovir treatment initially, and systemic steroids were introduced eight days later. He ultimately experienced significant clinical improvement.

## Introduction

Acute retinal necrosis (ARN) is a potentially sight-threatening disease characterized by anterior uveitis, vitritis, retinal vasculitis, and areas of necrotizing retinitis [1-6]. The diagnosis of ARN is based on clinical features including vitritis, occlusive retinal arteriolitis, and peripheral retinitis [1-3]. Furthermore, some patients with ARN develop the disease in the contralateral eye; this is called bilateral ARN (BARN) [1,2,4-6]. The varicella-zoster virus has been identified as the principal causative agent of ARN and BARN [2-6]. Additionally, immunosuppressive conditions, such as HIV, may be factors favoring the development of ARN and BARN [1]. Treatment options for managing these diseases include systemic antivirals, intravitreal antivirals, systemic corticosteroids, laser photocoagulation, and pars plana vitrectomy [2,3,5]. 

The use of systemic corticosteroids is controversial, as early initiation of corticosteroids may potentiate the rapid progression of retinitis by inducing viral replication [4, 5]. However, steroids may be used after the initiation of antivirals to manage specific inflammatory responses or complications associated with the treatment [5]. We present herein the case of a Hispanic male with BARN, whose disease was successfully managed with intravenous acyclovir, topical prednisolone, atropine, intravitreal ganciclovir, oral valacyclovir, and oral prednisone. The oral corticosteroid was added to his treatment regimen eight days after his initial presentation.

## Case presentation

A 55-year-old Hispanic male presented with a two-week history of bilateral blurred vision. At the time of presentation, the patient was being treated with prednisolone ophthalmic solution in both eyes (OU), every hour, and atropine 1% ophthalmic solution, three times daily, also OU. His medical history was remarkable for hypertension, obstructive sleep apnea, type two diabetes mellitus with peripheral neuropathy, remote varicella, and herpes zoster limited to the forehead. He had no previous ocular history. His review of systems and his social and family histories were otherwise unremarkable.

**Figure 1:**
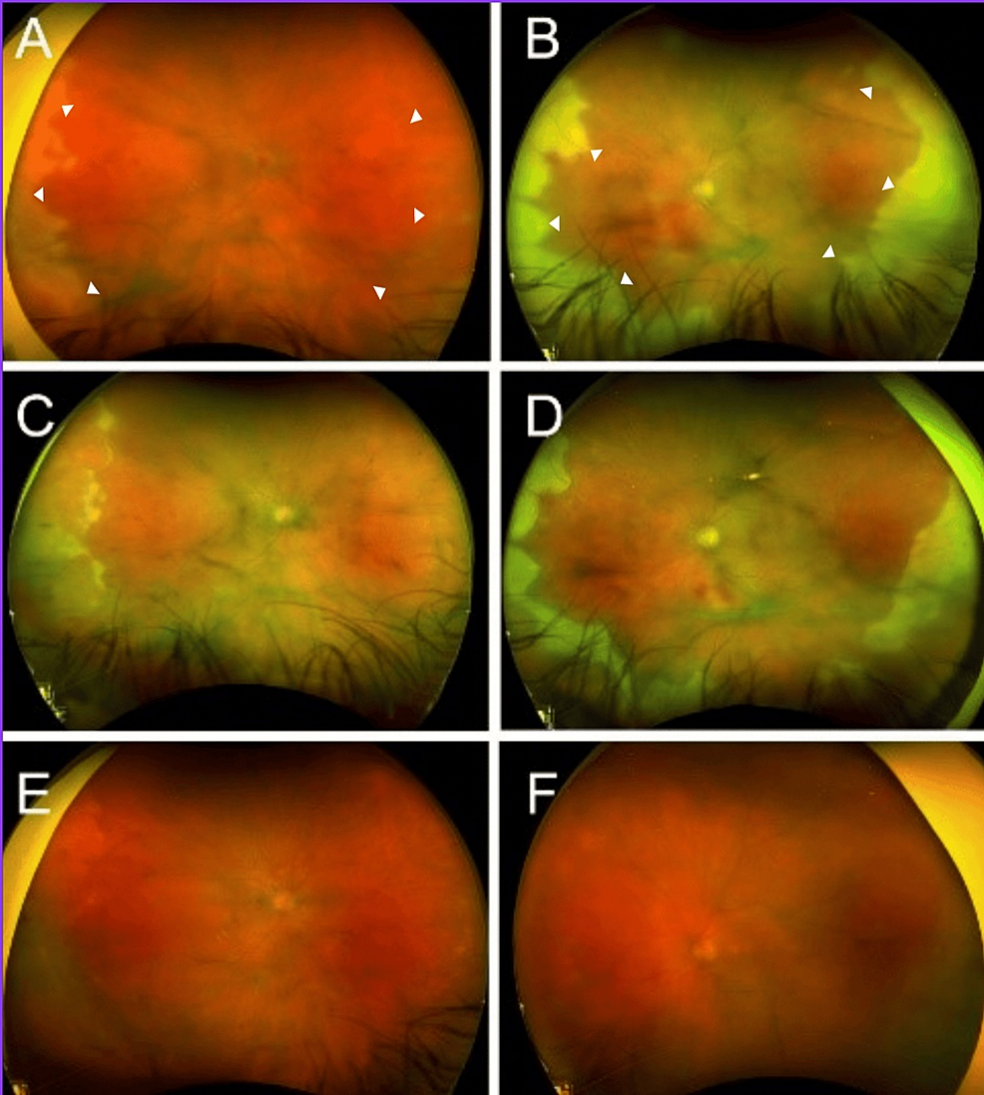
Serial color fundus photographs demonstrating treatment progression Initial presentation images of the right (A) and left (B) eyes reveal media opacities consistent with vitreous haze, right-sided disc hyperemia, and bilateral blurred disc margins. Additionally, focal retinitis (white arrowheads) is evident in both the nasal and temporal anterior peripheries. Follow-up images of the right (C) and left (D) eyes taken one week after initiating oral prednisone therapy showcase defined optic disc margins, a significant reduction in right disc hyperemia, and substantial improvement in areas previously affected by focal retinitis. Long-term follow-up images of the right (E) and left (F) eyes were captured five months post-initial presentation, indicating complete resolution of retinitis.

Upon a comprehensive ophthalmic evaluation, his best-corrected visual acuity (BCVA) was counting fingers (CF) OU. The intraocular pressure was 9 mmHg in OU. The pupils were round and pharmacologically dilated; therefore, the presence of an afferent pupillary defect could not be assessed. In neither eye could color vision or the visual field (using the confrontation visual field test) be assessed because of the patient's poor vision. Extraocular movements were within normal limits in OU. A slit-lamp examination revealed OU inferior scleral show, grade 3+ cells, pharmacologically dilated pupils, and grade 2+ vitreous cells (using the SUN classification). Examination of the patient's fundus revealed vitreous haze, right-sided disc hyperemia, bilateral blurred disc margins, and focal retinitis involving both the nasal and temporal anterior peripheries OU (Figures 1A, 1B).

The team made an assessment of BARN. Subsequently, the patient was admitted for intravenous therapy. He was started on 1,200 mg of acyclovir (calculated at 11 mg/kg), administered every eight hours, and instructed to continue the use of prednisolone ophthalmic solution every hour and atropine 1% ophthalmic solution three times daily OU. At this moment, the patient was injected with intravitreal ganciclovir, 2 mg, OU, with a second dose administered 48 hours later. Furthermore, an anterior chamber paracentesis was performed to obtain a sample for herpes simplex virus (HSV) 1 and 2 antibodies, varicella zoster virus (VZV) antibodies, and cytomegalovirus (CMV) antibodies. Notably, the test results indicated a positive finding for VZV antibodies, while HSV1, HSV2, and CMV antibodies were all detected as negative.

Eight days following treatment, his BCVA was 20/200 OU. A slit-lamp examination revealed fine keratic precipitates, grade 2+ cells in the anterior chamber, and grade 2+ cells in the vitreous OU. His dilated fundus examination remained similar to the initial exam OU. The patient opted to be discharged from the hospital against medical advice. He was prescribed oral prednisone and oral valacyclovir upon discharge. Specifically, prednisone was prescribed to be taken at a dosage of 60 mg daily for one week, followed by 40 mg daily for the subsequent week, and then it was to be stopped entirely. Valacyclovir was initially prescribed at 2 g taken orally three times daily and was continued at this dosage for two months. After this period, the dose was reduced to 1 g taken orally three times daily. The plan was to continue the valacyclovir indefinitely, eventually tapering to a maintenance dose of 1 g per day. One week after starting this regimen, his BCVA had improved to 20/70 in the right eye (OD) and 20/60 in the left eye (OS). A slit-lamp examination was unremarkable in OU. A dilated fundus examination revealed a significant reduction in right disc hyperemia and substantial improvement in areas previously affected by focal retinitis OU (Figures 1C, 1D). Furthermore, five months after the initial presentation, the patient had a BCVA of 20/40 in OU, and complete resolution of retinitis was evident on fundus examination (Figures 1E, 1F). The patient was advised to continue monthly follow-up for the following year.

## Discussion

Treating infection-mediated diseases such as BARN with systemic corticosteroids may seem controversial, as these medications can potentiate viral replication and the progression of retinitis [5]. Additionally, the use of systemic corticosteroids has been associated with the development of BARN [4,6]. It may therefore seem counterintuitive to treat these patients with systemic corticosteroids; nevertheless, timing is possibly the most vital factor for the successful management of this kind of infection. In the case presented, the patient received acyclovir treatment initially; systemic steroids were introduced eight days later, and he ultimately displayed significant clinical improvement.

Inducing a severe inflammatory reaction, BARN is characterized by anterior uveitis, vitritis, and necrotizing obliterative vasculitis [2,3,5]. Most reported cases describe acyclovir use in ARN and BARN to inhibit viral replication [3,5]. Moreover, the benefit of corticosteroid therapy is the suppression of the immune response, stabilization of blood vessels, reduction of vasodilation, and inhibition of inflammatory mediators, including cytokines and prostaglandins [5,7]. Corticosteroids therefore directly target vitritis and vasculitis, which are components of ARN [7]. Furthermore, steroid therapy has been suggested to confer the advantage of reducing the risk of secondary retinal detachment in cases of ARN [5].

It is important to note that this patient displayed a rare instance of BARN, as both of his eyes were affected simultaneously. Most reports describe a pattern in which one eye is affected first and the contralateral eye experiences the condition months to years later [3,5]. The choice of appropriate management may therefore vary greatly depending on each patient’s unique presentation. This case supports the use of corticosteroid therapy for the management of BARN; nevertheless, the potential risk of disease exacerbation necessitates prudent administration and vigilant monitoring.

## Conclusions

It is rare for BARN to afflict both eyes simultaneously, and the potential for disease exacerbation emphasizes the need for individualized management strategies. This case suggests that patients with BARN may benefit from the use of systemic corticosteroids. Nevertheless, timing is possibly the most vital factor for the successful management of BARN and the avoidance of disease exacerbation.
